# G-CSF upregulates the expression of aquaporin-9 through CEBPB to enhance the cytotoxic activity of arsenic trioxide to acute myeloid leukemia cells

**DOI:** 10.1186/s12935-022-02613-y

**Published:** 2022-05-19

**Authors:** Wanbin Fu, Gelan Zhu, Lan Xu, Jia Liu, Xiaofeng Han, Junying Wang, Xinpeng Wang, Jian Hou, Huanbin Zhao, Hua Zhong

**Affiliations:** 1 Department of Hematology, Ren Ji Hospital, Shanghai Jiao Tong University School of Medicine, Shanghai, China; 2 Department of Pathophysiology, Key Laboratory of Cell Differentiation and Apoptosis of Chinese Ministry of Education, Shanghai Jiao Tong University School of Medicine, Shanghai, China

**Keywords:** Acute myeloid leukemia, Arsenic trioxide, G-CSF, AQP9, CEBPB

## Abstract

**Background:**

Arsenic trioxide (ATO) is highly effective in acute promyelocytic leukemia (APL) patients, but it fails to show satisfactory efficacy in other acute myeloid leukemia (AML) patients with non-APL subtypes. Different from the APL cells, most non-APL AML cells express low levels of the ATO transporter Aquaporin-9 (AQP9) protein, making them less sensitive to ATO treatment. Recently, we found that granulocyte colony stimulating factor (G-CSF) can upregulate the expression of AQP9. We hypothesized that the pretreatment with G-CSF may enhance the antitumor effect of ATO in non-APL AML cells. In addition, we aimed to elucidate the underlying mechanisms by which G-CSF upregulates the expression of AQP9.

**Methods:**

Non-APL AML cell lines including THP-1 and HL-60 were pretreated with or without G-CSF (100 ng/ml) for 24 h, followed by the treatment with ATO (2 μM) for 48 h. Cell morphology was observed under the microscope after Wright-Giemsa staining. Flow cytometry was performed to evaluate the cell apoptosis levels. The intracellular concentrations of ATO were determined by atomic fluorescence spectrometry. The mRNA and protein expression were respectively measured by quantitative reverse transcription PCR (RT-qPCR) and western blotting. Target genes were knocked down by transfection with small interfering RNA (siRNA), or overexpressed by transfection with overexpression plasmids. The cell line derived xenograft mouse model was established to confirm the results of the in vitro experiments.

**Results:**

Compared with using ATO alone, the combination of G-CSF with ATO induced the cell apoptosis more dramatically. G-CSF upregulated the expression of AQP9 and enhanced the intracellular concentrations of ATO in AML cells. When AQP9 was overexpressed, it markedly enhanced the cytotoxic activity of ATO. On the other hand, when AQP9 was knocked down, it profoundly attenuated the combinational effect. Moreover, we found that the upregulation of AQP9 by G-CSF depends on the transcription factor CCAAT enhancer binding protein beta (CEBPB). We also demonstrated that the combination of G-CSF and ATO significantly inhibited tumor growth in the xenograft mouse model.

**Conclusions:**

The combination of G-CSF and ATO may be a potential therapeutic strategy for AML patients.

## Background

Acute myeloid leukemia is an aggressive malignancy characterized by uncontrolled proliferation and accumulation of immature myeloid precursor cells in the bone marrow [1]. AML is a disease of the elderly, with a median age of 68 years old at diagnosis [2]. For older unfit patients, the traditional remission induction regimen may bring high treatment-related mortality, and little benefit to overall survival time. New therapeutic options including demethylating agents and BCL-2 inhibitors lead to a slight increase in life expectancy, but the prognosis remains poor in older patients (5-year overall survival rates of < 20% and < 5% among patients aged 65–74 and > 75, respectively) [3]. Finding appropriate drugs for these frailer patients is a major challenge in the treatment of AML.

ATO has been applied in APL (AML-M3, a specific type of AML) treatment for decades and has achieved satisfactory therapeutic effects [4, 5]. ATO can target and degrade the promyelocytic leukemia-retinoic acid receptor-α (PML/RARα) protein, which is the abnormal protein product generated by APL-specific t(15;17) chromosomal translocation and is essential for the leukemogenesis of APL [4, 5]. ATO was also tested in non-APL AML patients in serveral clinical trials, combined with subcutaneous low-dose cytarabine or with ascorbic acid and decitabine [6]. However, it seemed that the addition of ATO did not bring more benefits to those patients [6]. It has been reported that the uptake of ATO into leukemia cells depends on AQP9, a transmembrane transporter belonging to the aquaporin family [7, 8]. Different from the APL cells, most non-APL AML cells express low levels of AQP9 proteins [7]. As a result, they show less sensitivity to ATO treatment. Improving the expression of AQP9 is the key point to overcoming ATO resistance in acute myeloid leukemia. The mechanisms of how AQP9 is regulated in leukemia cells still remain not very clear. It was reported that the expression of AQP9 can be regulated by HNF1 homeobox A (HNF1A) [9]. Demethylating the promoter region of *HNF1A* gene can increase the expression of the HNF1A proteins, which can ultimately upregulate the protein level of AQP9 [9]. It has also been reported that the phosphorylation of p38 MAPK signaling pathways can promote the expression of AQP9 in rat brains or astrocytes [10, 11].

Granulocyte colony stimulating factor is important for the differentiation and proliferation of normal myeloid precursors [12]. G-CSF can bind with its receptor to form a dimer, resulting in the activation of the downstream signaling pathways including JAK/STAT, Src kinases, Lyn, Ras/ERK (MAPK signaling pathway), and PI3K [13]. G-CSF can also enhance the cytotoxic effect of chemotherapeutic drugs in the prime protocol of acute myeloid leukemia [14].

In this study, we hypothesize that G-CSF can increase the sensitivity of leukemia cells to ATO by upregulating the protein expression of AQP9. On this basis, we will further elucidate the mechanisms of how AQP9 is regulated by G-CSF.

## Methods

### Cell culture and treatment of leukemia cells

The acute myeloid leukemia cell lines, including HL-60 (non-APL AML-derived), THP-1 (non-APL AML-derived), and NB4 (APL-derived), were purchased from the Cell Bank of the Chinese Academy of Sciences. The cells were cultured in RPMI-1640 (Corning, 10-040-CVRC) supplemented with 10% fetal bovine serum (GIBCO) in the cell incubator with 5% CO_2_ at 37 °C.

1 × 10^6^ cells of each group were pretreated with or without G-CSF (100 ng/ml) for 24 h, and after that cells were treated with or without ATO (2 μM) for 48 h. Then 100 μl cells of each group with gradient concentrations (2 × 10^5^/ml, 1 × 10^5^/ml, and 5 × 10^4^/ml) were centrifuged onto glass slides at 500 rpm for 5 min. The slides were stained by Wright-Giemsa staining solution (Beyotime, C0135), and then were observed or photographed under the microscope.

### Flow cytometry analysis of cell apoptosis

To analyze the apoptosis levels of cells, THP-1 or HL-60 cells were pretreated with or without G-CSF (100 ng/ml, dissolved in saline solution) for 24 h, followed by the treatment with or without ATO (2 μM, diluted in saline solution) for 48 h. 2 × 10^6^ cells were collected and washed with PBS buffer once and with binding buffer (ThermoFisher) once again. Then the cells were resuspended by 100 μl binding buffer. Each sample was stained by 5 μl Annexin V (ThermoFisher) for 15 min at room temperature in the dark. Then the cell samples were washed with 2 ml binding buffer and resuspended by 200 μl binding buffer. The cell suspension was added with 5 μl propidium iodide (Invitrogen) to stain for 10 min before analysis by flow cytometry (BD).

### Western blotting

To prepare protein samples, 2 × 10^6^ cells of each sample were collected and washed by PBS once. Each sample was lysed by 1 × SDS loading buffer (Beyotime, P0015) and boiled at 100 °C. Then protein samples were separated by SDS–polyacrylamide gel electrophoresis and transferred onto nitrocellulose membrane (0.22 μm). The antibodies and the usage concentrations are as follows: anti-AQP9 antibody (Alpha Diagnostic International, 1:1000), anti-CEBPB antibody (Santa Cruz, 1:1000), anti-GAPDH antibody (Proteintech, 1:2000), HRP-linked anti-Rabbit IgG antibody (Santa Cruz, 1:2000).

### RT-qPCR

For RT-qPCR analysis, 4 × 10^6^ cells of each sample were collected and washed by PBS once. Each sample was lysed by TRIZOL (Sigma). RNA was extracted and reversely transcribed as protocol. The transcriptional levels of each gene were analyzed on the 7500 Fast Real-Time PCR System. The primers of the target genes and the reference gene (*GAPDH*) are as follows (all the sequences are from 5′ to 3′):

*GAPDH*-F, AGAAGGCTGGGGCTCATT,

*GAPDH*-R, TGCTAAGCAGTTGGTGG;

*AQP9*-F, TGCTGGTGGAAAACTGCTGA,

*AQP9*-R, TTCCAGCTTTGATCTGCAAATGCG;

*CEBPB*-F, CTTCAGCCCGTACCTGGAG,

*CEBPB*-R, GGAGAGGAAGTCGTGGTGC;

*FOS*-F, GGGGCAAGGTGGAACAGTTAT,

*FOS*-R, CCGCTTGGAGTGTATCAGTCA;

*NFKB1*-F, GAAGCACGAATGACAGAGGC,

*NFKB1*-R, GCTTGGCGGATTAGCTCTTTT;

*STAT3*-F, ACCAGCAGTATAGCCGCTTC,

*STAT3*-R, GCCACAATCCGGGCAATCT;

*SP1*-F, TGGCAGCAGTACCAATGGC,

*SP1*-R, CCAGGTAGTCCTGTCAGAACTT.

### Cell transfection

The THP-1 or HL-60 cells in the logarithmic growth phase were transfected with siRNAs or plasmids. The siRNA targets of the *AQP9* gene and the *CEBPB* gene are as follows (all the sequences are from 5′ to 3′):

si-*AQP9*-92, GUCAAAACGUCCAUUUUCA;

si-*AQP9*-142, GUAGGUAUUGGUAGAAACA;

si-*AQP9*-480, GUUCAAAUUGCCAUUUUAU;

si-*CEBPB*-992, CCAAGAAGACCGUGGACAA;

si-*CEBPB*-1010, AGCACAGCGACGAGUACAA;

si-*CEBPB*-1030, AGAACGAGCGGCUGCAGAA;

Negative control, UUCUCCGAACGUGUCACGU.

### The atomic fluorescence spectrometry

THP-1 or HL-60 cells were pretreated with or without G-CSF (100 ng/ml) for 24 h, and then were treated with ATO (2 μM) for 48 h. After that, 5 × 10^6^ cells were collected and broken up by ultrasonication. The cell debris was dissolved by test solution (66.7% HNO_3_ + 33.3% H_2_O_2_) and was analyzed for the concentrations of atomic arsenic by Agilent 7800 ICP-MS.

### The cell line derived xenograft mouse model and drug administration

The protocol of the establishment of xenograft mouse model referred to the previous studies [15, 16]. Approximately 6–8 weeks old female BALB/c athymic nude mice (immunocompromised in T cell development) were used for the xenograft animal model. To establish the xenograft mouse model of AML cells, THP-1 cells (1 × 10^7^ cells per mouse) were subcutaneously injected into the dorsal of the mice under brief anesthesia by intraperitoneal injection of tribromoethyl alcohol (350 mg/kg). And then those mice bearing THP-1 cells were administered with ATO alone (2 mg/kg) or ATO/G-CSF (G-CSF 300 μg/kg, ATO 2 mg/kg), or the saline solution as the vehicle control, by peritoneal injection once a day when the tumor volumes reached 100 mm^3^. The number of the mice for each group was ≥ 5. The tumor volumes were recorded every 3 days. The tumor volume was calculated by the following formula: (length × width^2^)/2. All the mice were euthanized by intraperitoneal injection of pentobarbital (200 mg/kg) on Day 17 after treatment, and the carefully separated tumors were photographed and weighted. All the animal experiments were approved and supervised by the Medical Ethics Committee of Ren Ji Hospital affiliated to Shanghai Jiao Tong University School of Medicine.

### Immunohistochemistry and HE staining

The procedures of immunohistochemistry are as follows. The tumor tissues were carefully separated, fixed in 4% paraformaldehyde, and embedded in paraffin. The embedded tissues were sliced into sections. The sections went through deparaffinization, rehydration, and antigen retrieval. Then the sections were blotted with the primary antibodies against AQP9 (Alpha Diagnostic International) or CEBPB (Santa Cruz). After wash steps by PBS, the sections were blotted by the biotin-associated secondary antibody. The sections were developed with the substrate mixture.

As for the analysis of mouse livers, the liver tissues were carefully separated from the nude mice receiving ATO or G-CSF/ATO treatment. The tissue sections were prepared for the immunohistochemistry procedures. The sections were stained with hematoxylin and eosin (HE) using the HE Staining Kit (Beyotime, C0105S). All the procedures were performed as protocol. All the finished sections were observed and photographed under the microscope.

### Statistical analysis

The data in this study were all analyzed and presented using Graphpad Prism software (version 8) or Excel software. The apoptosis levels of cells, the concentrations of ATO, or the weights of tumors from different groups were statistically analyzed by unpaired student t-test. P < 0.05 represents the significant difference between the different groups. NS represents no significant difference.

## Results

### G-CSF significantly promotes the sensitivity of AML cells to ATO

**Fig. 1:**
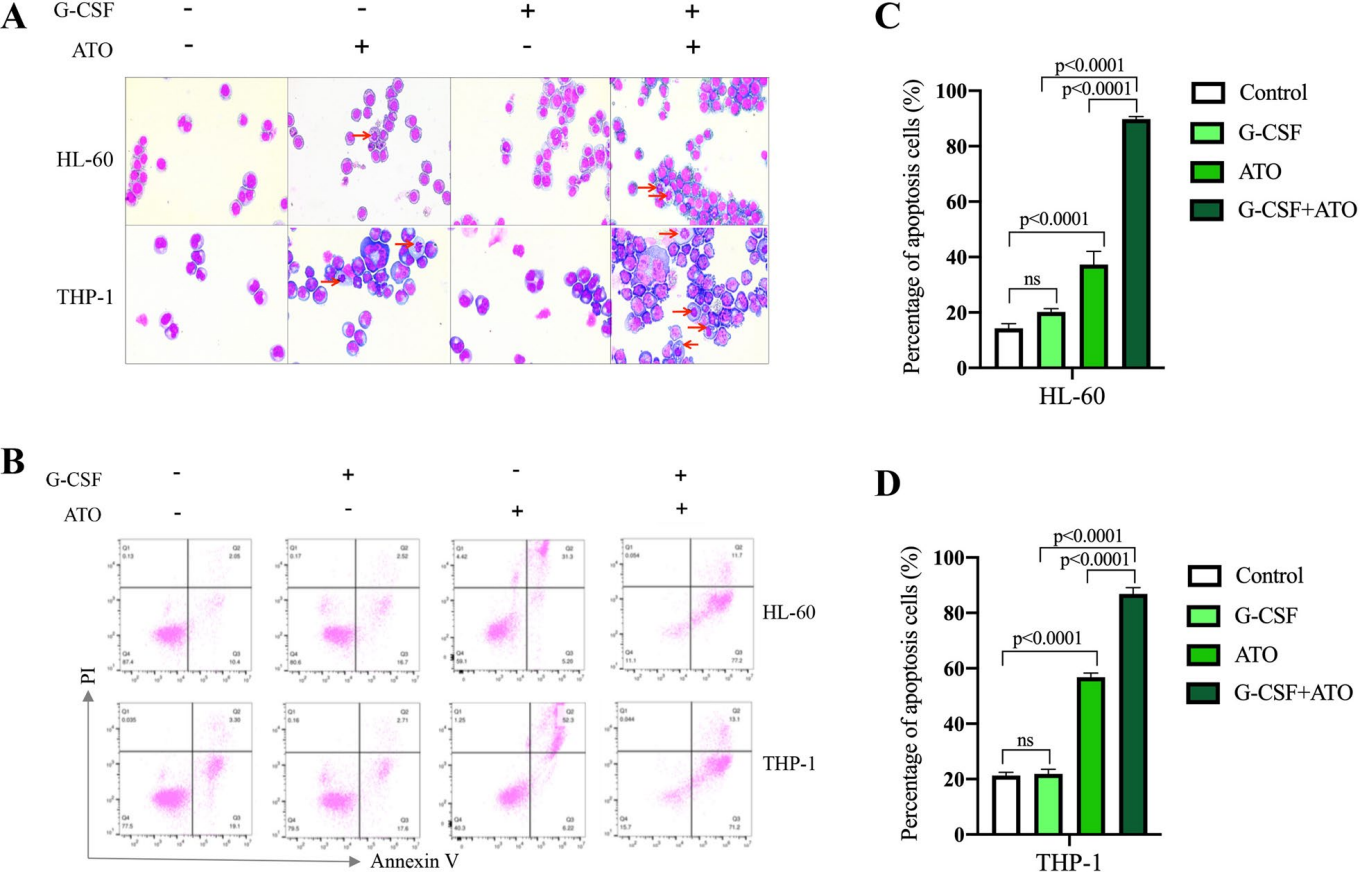
G-CSF promotes the sensitivity of AML cells to ATO. **A** The morphology of HL-60 and THP-1 cells after the indicated treatment. **B** The apoptosis levels of the indicated groups were examined by flow cytometry. **C**, **D** The statistical analysis of the apoptosis levels of HL-60 (**C**) and THP-1 (**D**) cells after the indicated treatment

To compare the effects of ATO alone and ATO/G-CSF combination on AML cells, we treated THP-1 and HL-60 cells with or without G-CSF for 24 h, followed by the treatment with or without ATO for 48 h. We found that the cells treated with G-CSF didn’t exhibit remarkable morphological change compared with the control cells (Fig. 1A, lane 1–2), while a small portion of cells underwent apoptosis when treated with ATO alone (Fig. 1A, lane 3). In contrast, the number of apoptotic cells significantly increased in the combination group (Fig. 1A, lane 4). We further used flow cytometry to analyze the apoptosis levels. As the observations above, the treatment with ATO alone slightly induced cell apoptosis, while the combination of G-CSF and ATO greatly increased the apoptosis levels (Fig. 1B–D). These results indicate that G-CSF can promote the cytotoxic effect of ATO.

### G-CSF upregulates the expression of AQP9 and enhances the intracellular concentrations of ATO in AML cells

**Fig. 2:**
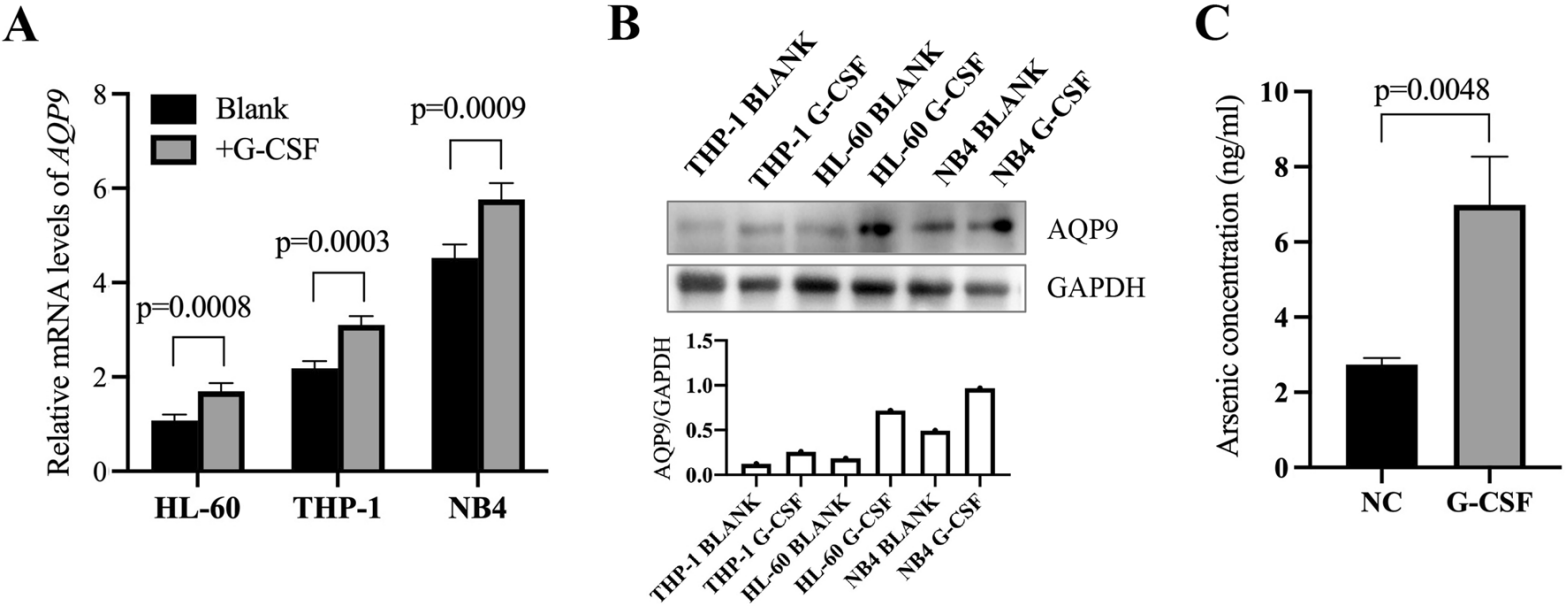
G-CSF upregulates the expression of AQP9 and the ATO concentrations in AML cells. **A**, **B** The mRNA and protein levels of AQP9 of the indicated groups were examined. **C** The ATO concentrations of HL-60 cells after the indicated treatment were examined by atomic fluorescence spectrometry

Since AQP9 was reported to be the key membrane channel for ATO uptake [17, 18], we hypothesized that G-CSF enhanced the cytotoxic effect of ATO by upregulating the expression of AQP9. We treated the cells (THP-1, HL-60, and NB4) with G-CSF and collected the cells for RT-qPCR and western blotting analysis. Among those cell lines, NB4 is derived from APL, while HL-60 and THP-1 are derived from non-APL AML. Firstly, we found that the expression of AQP9 was significantly higher in NB4 cells than in HL-60 and THP-1 cells (Fig. 2A and B). Moreover, we found that the expression of AQP9 was upregulated after treatment with G-CSF (Fig. 2A and B). We also examined the intracellular concentrations of ATO by atomic fluorescence spectrometry and found that the ATO concentrations increased after treatment with G-CSF (Fig. 2C).

### AQP9 is required for the transportation of ATO in AML cells

**Fig. 3:**
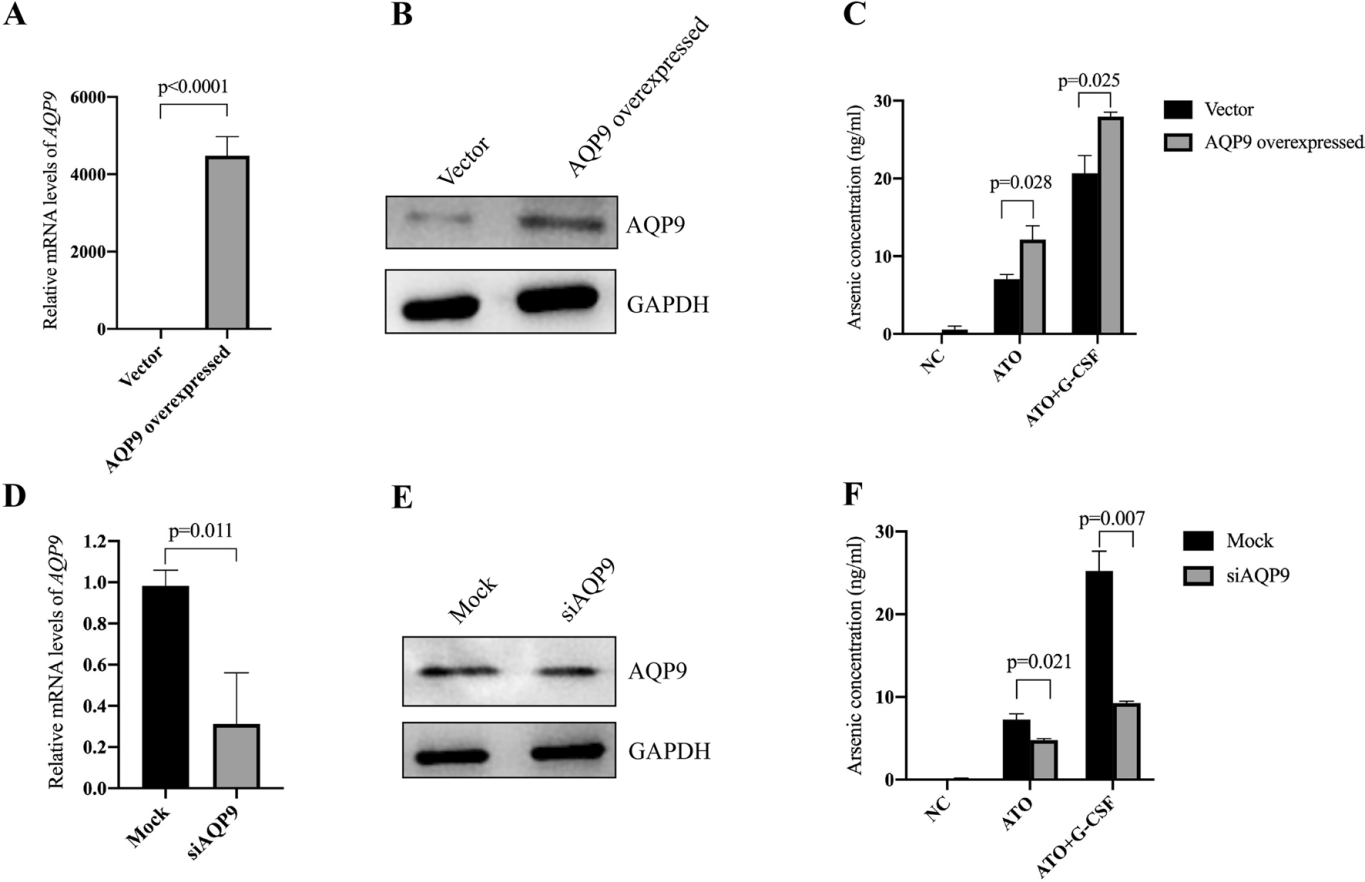
AQP9 is required for the transportation of ATO in AML cells. **A**–**C** The mRNA levels (**A**) and protein levels (**B**) of THP-1 cells overexpressed with AQP9 were examined, and the ATO concentrations were examined after the indicated treatment (**C**). **D**–**F** The mRNA levels (**D**) and protein levels (**E**) of THP-1 cells transfected with siRNA targeting AQP9 were examined, and the ATO concentrations were examined after the indicated treatment (**F**)

To further confirm whether AQP9 is required for the transportation of ATO in AML cells, we firstly overexpressed AQP9 in THP-1 cells (Fig. 3A and B). After treatment with ATO alone or G-CSF/ATO, the intracellular concentrations of ATO were examined. It was found that the ATO concentrations accumulated much more in the overexpression group than in the vector group (Fig. 3C). We also interfered the expression of AQP9 by siRNA (Fig. 3D and E). In contrast to the results of the overexpression experiments, the knockdown of AQP9 remarkably reduced the ATO concentrations (Fig. 3F). These results suggest that AQP9 is required for the transportation of ATO in AML cells.

### AQP9 is essential for the combinational effect of G-CSF and ATO

**Fig. 4:**
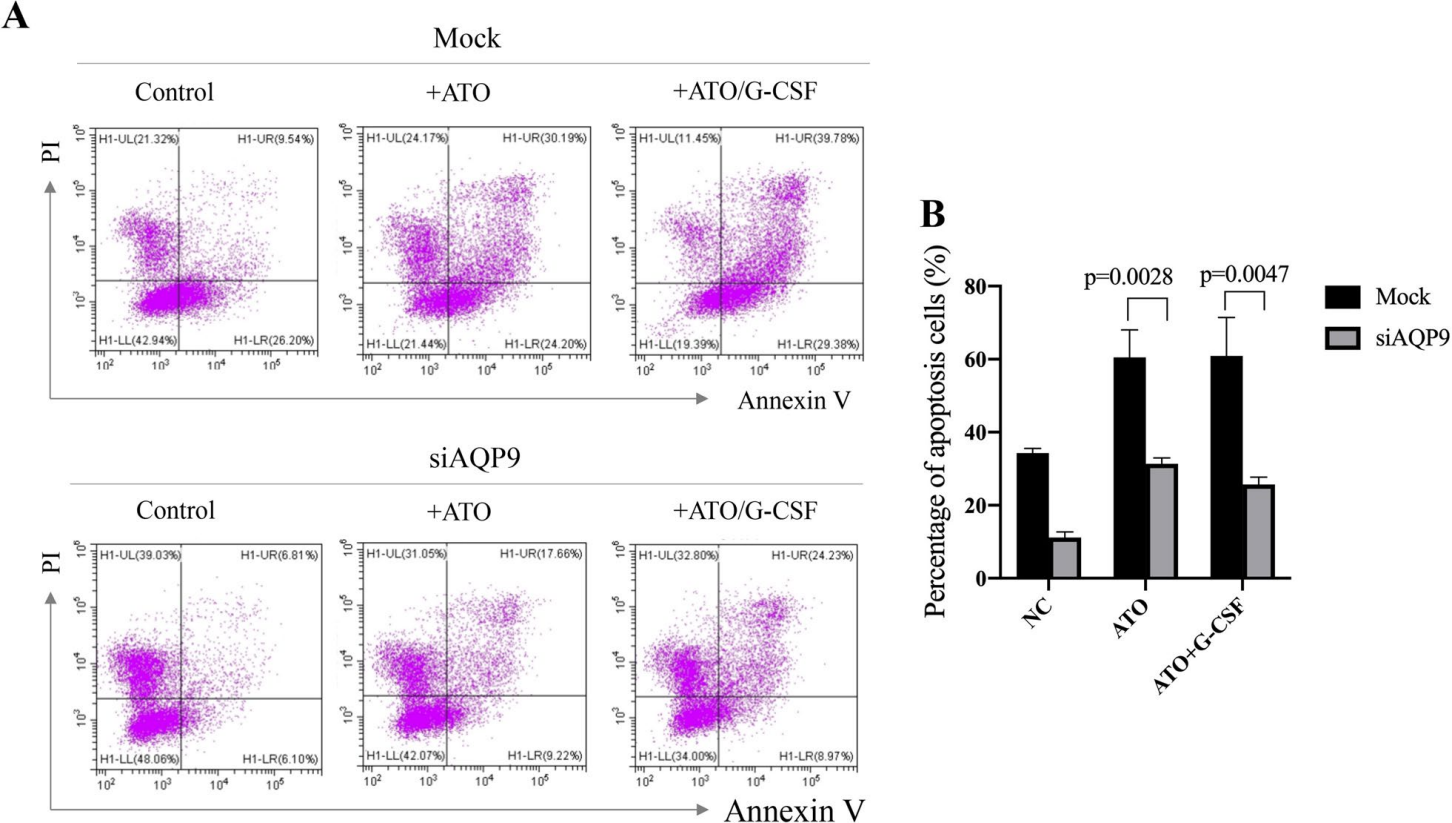
AQP9 contributes to the combinational effect of G-CSF and ATO. The apoptosis levels of the indicated cells were examined by flow cytometry (**A**) and statistically analyzed (**B**)

To investigate the role of AQP9 in the combinational effect of G-CSF and ATO on AML cells, we interfered the expression of AQP9 by siRNA in THP-1 cells (mock siRNA as negative control) and then treated with the cells with ATO alone or G-CSF/ATO in combination (the vehicles as control). And then the apoptosis levels were analyzed. We found that the apoptosis induction ability of ATO was significantly inhibited after AQP9 knockdown (p = 0.0028, Fig. 4A and B), while the combinational effect of G-CSF and ATO was also dramatically suppressed after knockdown of AQP9 (p = 0.0047, Fig. 4A and B), suggesting that AQP9 is essential for the combinational effect.

### The upregulation of AQP9 by G-CSF depends on CEBPB

**Fig. 5:**
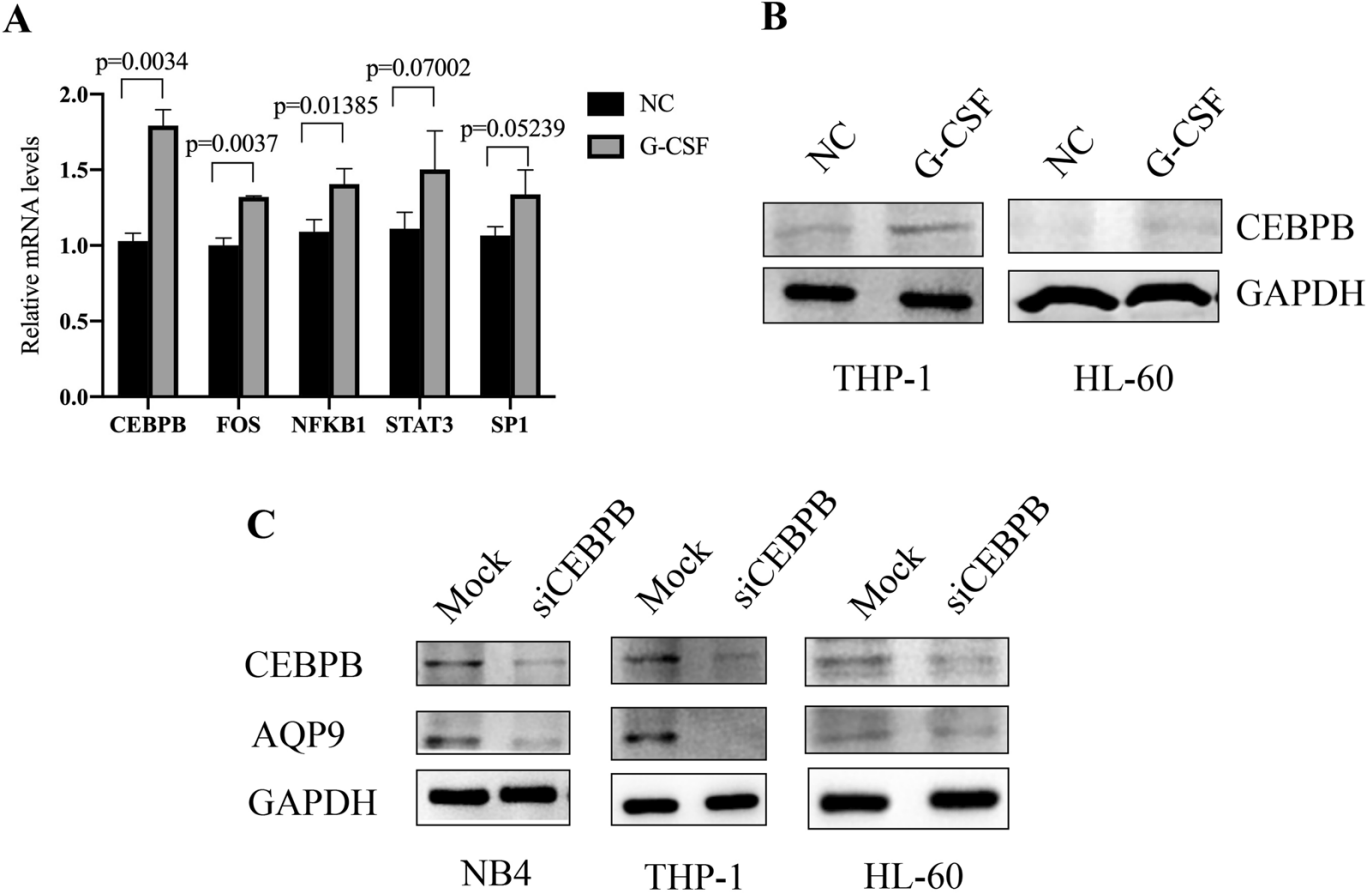
G-CSF upregulates the expression of AQP9 through CEBPB. **A** The mRNA levels of the indicated transcription factors in HL-60 cells were examined by RT-qPCR after treatment with or without G-CSF for 24 h. **B** The protein levels of CEBPB in HL-60 and THP-1 cells were examined by western blotting after treatment with or without G-CSF for 24 h. **C** The protein levels of AQP9 and CEBPB in the three cell lines transfected with siRNA targeting CEBPB or mock siRNA were examined by western blotting

To explore the mechanisms of how G-CSF upregulated the expression of AQP9, we examined the transcriptional expression of several potential transcription factors including CEBPB, FOS, NFKB1, STAT3, and SP1 by RT-qPCR. We found that the mRNA levels of the *CEBPB* gene were upregulated most significantly (p = 0.0034, Fig. 5A). We further examined the expression of CEBPB by western blotting, and found that G-CSF indeed remarkably promoted the protein levels of CEBPB in THP-1 and HL-60 cells (Fig. 5B). We also knocked down *CEBPB* gene and then examined the expression of AQP9. It was found that the inhibition of CEBPB could markedly suppress the expression of AQP9 (Fig. 5C), suggesting that CEBPB is necessary for the upregulation of AQP9 in response to G-CSF.

### The combination of G-CSF and ATO suppressed the growth of AML cells in the xenograft mouse model

**Fig. 6:**
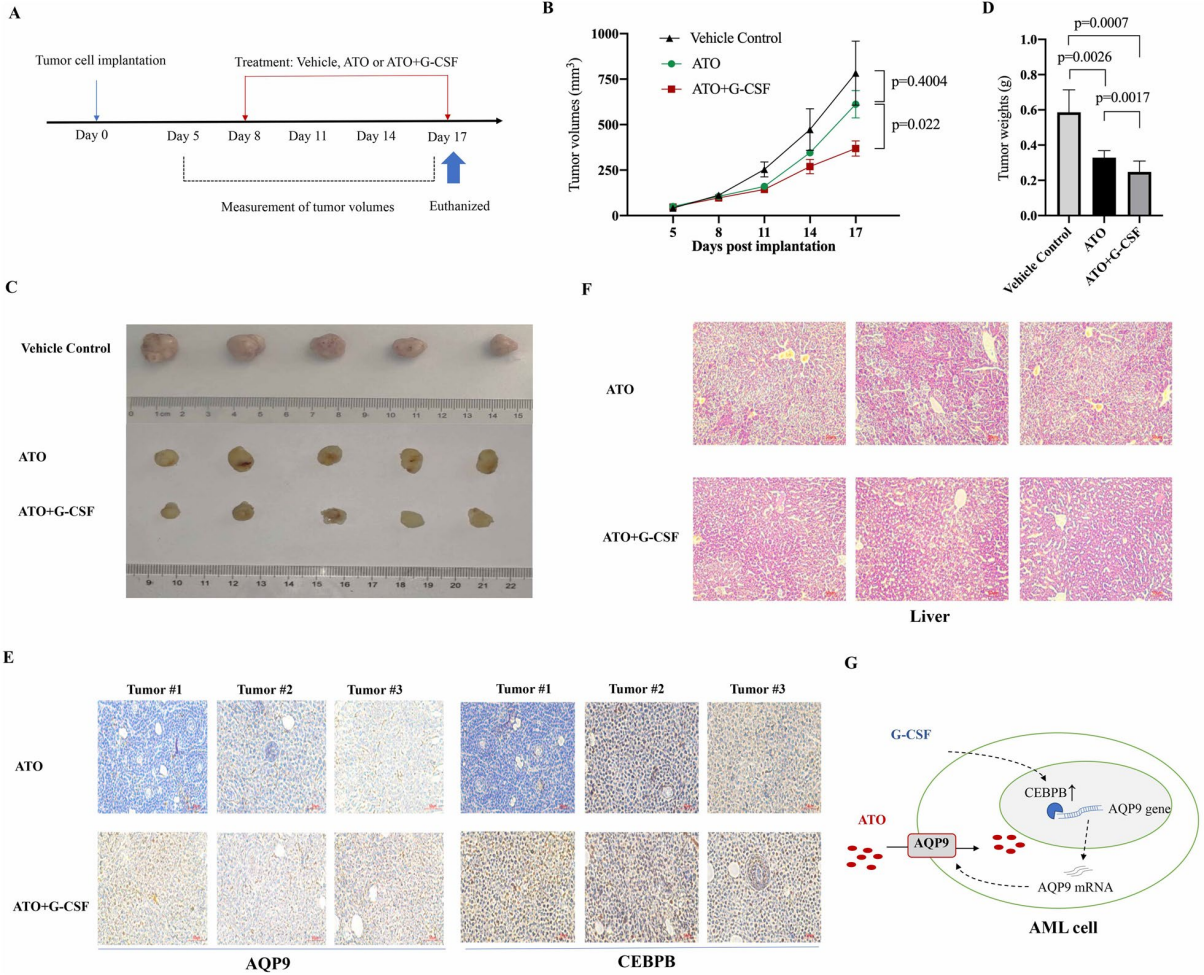
The combination of G-CSF and ATO inhibited the tumor growth of AML cells in vivo. **A** The scheme for the establishment of the xenograft mouse model and drug treatment. **B** The tumor growth curves of the indicated groups. **C**, **D** The mice were euthanized on Day 17 after treatment, and the tumors were separated and weighted. **E** Immunohistochemistry with anti-CEBPB and anti-AQP9 antibodies for the tumor sections from the indicated groups. **F** The HE staining of liver sections from the indicated groups. **G** The hypothetical mechanism of the combinational effect of G-CSF and ATO

To determine the effect of ATO/G-CSF in vivo, THP-1 cells were subcutaneously injected into nude mice to establish the xenograft mouse model. And then those mice bearing THP-1 cells were administered with ATO alone or ATO/G-CSF in combination. The saline solution was injected in another group of mice as the vehicle control. The tumor volumes were recorded every 3 days (Fig. 6A). We found that the growth of the tumors was significantly slower in the ATO/G-CSF group, compared with the ATO group and the vehicle control group (Fig. 6B). All the mice were euthanized on Day 17 after treatment and the tumors were carefully separated. It was shown that the tumors of the G-CSF/ATO group were significantly smaller than the vehicle control group and the ATO group (p = 0.0007 and p = 0.0017, Fig. 6C and D). We also examined the expression of CEBPB and AQP9 in the tumor tissues by immunohistochemistry. It was found that the expression of CEBPB and AQP9 was much stronger in the ATO/G-CSF group than in the ATO group (Fig. 6E), consistent with the observations in the previous in vitro experiments.

### G-CSF didn’t enhance the liver toxicity of ATO in the xenograft mouse model

Since ATO may exhibit liver toxicity in clinical practice [19], we then tested whether G-CSF would aggravate liver dysfunction by promoting the uptake of ATO. We separated the livers from the mice treated with ATO alone or G-CSF/ATO in combination, and analyzed the liver morphology after HE staining. We didn’t observe severe liver toxicity in the ATO group or the G-CSF/ATO group of mice, and there was no significant difference between the two groups of livers (Fig. 6F).

## Discussion

Arsenic trioxide is highly effective in recurrent and newly diagnosed acute promyelocytic leukemia. In AML patients of non-APL subtypes, some researchers tried to add ATO to the subcutaneous injection of low-dose cytarabine, hoping to improve the clinical treatment effect. Unfortunately, the addition of ATO didn’t show benefit for these AML patients [20]. Arsenic trioxide has anti-proliferative and pro-apoptotic effects on a variety of AML cell lines in vitro [21]. ATO affects the biological behavior of leukemia cells through a series of mechanisms in AML, including induction of cell cycle arrest, apoptosis, and autophagy; generation of reactive oxygen species and accumulation of intracellular hydrogen peroxide; release of cytochrome c and activation of caspases; inhibition of glutathione peroxidase; and promotion of differentiation and anti-angiogenesis [22–24]. Considering the antitumor potential of ATO and its manageable toxicities, it’s necessary to investigate other strategies to improve the clinical efficacy of ATO in non-APL AML patients.

G-CSF is an important cytokine that regulates cell growth, differentiation, and migration. It has been widely used in patients with neutropenia after chemotherapy. In this study, we demonstrated that the pretreatment with G-CSF can significantly promote the cytotoxic effect of ATO on AML cell lines. The combination of ATO and G-CSF can induce cell apoptosis dramatically. We also found that the combination of G-CSF with ATO can also increase the intracellular concentration of ATO in AML cells which can further inhibit cell proliferation and induce cell apoptosis. To evaluate the combinational effect of G-CSF and ATO more precisely, more experiments using the primary cells from AML patients or using the patient-derived xenograft models would be expected.

As a member of AQP families, AQP9 is the only channel that has been reported to be responsible for arsenic uptake [17, 18]. We and other researchers all found that the expression of AQP9 was higher in NB4 cells than in HL-60 and THP-1 cells [7, 18]. This may partly explain the reason why ATO is more sensitive in APL than non-M3 AML. Moreover, when we treated HL-60 or THP-1 cells with G-CSF, we found that the expression of AQP9 was significantly upregulated, and that the upregulation was abolished when AQP9 was knocked down by siRNA, suggesting that G-CSF indeed enhances the transportation of ATO through the regulation of AQP9. Liver dysfunction is the major side effect of ATO treatment in clinical practice [19]. We compared the liver morphology between the two groups of mice treated with ATO alone and G-CSF/ATO in combination. We found that the addition of G-CSF didn’t increase the obvious hepatic impairment rate. This finding is consistent with the results of a previous Phase I clinical trial that treated AML patients with the combination of ATO and decitabine [25], a demethylating agent that may also upregulate the expression of AQP9 [9].

Several potential transcription factors including CEBPB, FOS, NFKB1, STAT3, and SP1 may respond to G-CSF treatment. CEBPB plays an important role in hematopoiesis. It has been predicted that CEBPB is a potential transcript factor of AQP9 [26]. When CEBPB was knocked down, the expression of AQP9 was also dramatically inhibited. This suggests that the expression of AQP9 may be regulated by the transcription factor CEBPB. It was reported that JAK-STAT3 signal pathway is the upper regulator of CEBPB [27]. We propose that when G-CSF binds its receptor G-CSFR, the JAK-STAT3 signal pathway can be activated, resulting in the upregulation of CEBPB. More studies will be performed to reveal the mechanism of how CEBPB regulates the transcription of AQP9 in the future. In addition, since it has been reported that p38 can regulate the expression of AQP9 [10], and that p38α can regulate the transcription of CEBPB [27], the role of p38 in the G-CSF-CEBPB-AQP9 axis also remains to be characterized.

## Conclusions

In conclusion, we demonstrate that the pretreatment with G-CSF can significantly enhance the cytotoxic activity of ATO by upregulating the expression of AQP9 in non-APL AML cells in vitro and in vivo (as shown in Fig. 6G). The combination of G-CSF and ATO would be a potential therapeutic strategy for AML patients.

## Data Availability

Not applicable.

## Abbreviations

G-CSF: Granulocyte colony stimulating factor
ATO: Arsenic trioxide
AML: Acute myeloid leukemia
APL: Acute promyelocytic leukemia
PML/RARα: Promyelocytic leukemia-retinoic acid receptor-α
AQP9: Aquaporin-9
CEBPB: CCAAT enhancer binding protein beta
RT-qPCR: Quantitative reverse transcription PCR
siRNA: Small interfering RNA
HNF1A: HNF1 homeobox A
MAPK: Mitogen-activated protein kinase
NC: Negative control
HE: Hematoxylin and eosin
